# A novel AIRE mutation leads to autoimmune polyendocrine syndrome type-1

**DOI:** 10.3389/fcell.2022.948350

**Published:** 2022-08-22

**Authors:** Guofeng Qian, Xiaoyi Yan, Junli Xuan, Danfeng Zheng, Zhiwen He, Jianguo Shen

**Affiliations:** ^1^ Department of Endocrinology, The First Affiliated Hospital, College of Medicine, Zhejiang University, Hangzhou, China; ^2^ Department of Cell Biology, College of Medicine, Zhejiang University, Hangzhou, China; ^3^ Imaging Facility of Core Facilities, College of Medicine, Zhejiang University, Hangzhou, China; ^4^ Department of Laboratory Medicine, The First Affiliated Hospital, College of Medicine, Zhejiang University, Hangzhou, China; ^5^ Department of Colorectal and Anal Surgery, The First Affiliated Hospital, College of Medicine, Zhejiang University, Hangzhou, China

**Keywords:** autoimmune polyendocrine syndrome type-1 (APS-1), autoimmune regulator (AIRE), homozygous mutation, exon, pedigree

## Abstract

Autoimmune polyendocrine syndrome type-1 (APS-1) is a rare inherited monogenic autoimmune disease characterized by the presence of at least two of three following major clinical features: chronic mucocutaneous candidiasis, hypoparathyroidism, and adrenal insufficiency. Mutations in autoimmune regulator (AIRE) gene have been found to contribute to APS-1. In the present study, we reported a 36-years-old male APS-1 patient who presented with hypoparathyroidism and Addison’s disease. The proband underwent complete clinical examinations and mutation screening was performed by Sanger sequencing on AIRE gene. A novel homozygous mutation in exon 9 of the AIRE gene (c.1024C>T) was identified. Based on sequencing findings, HEK293T cell-based assays were conducted to analyze the subcellular localization and mutant transcript processing. Our results revealed that p.Q342X mutant localized in nuclear speckles and exerted a dominant-negative effect on wildtype AIRE function. We reported the c.1024C>T mutation of AIRE gene for the first time, which enriched the AIRE mutation database and contributed to further understanding of APS-1.

## 1 Introduction

Autoimmune polyendocrine syndrome type-1 (APS-1) is a rare inherited monogenic autoimmune disease characterized by the presence of at least two of the three following major clinical features: chronic mucocutaneous candidiasis, hypoparathyroidism, and adrenal insufficiency (Perheentupa, 2006). Mutations in autoimmune regulator (AIRE) have been found to contribute to APS-1 (Finnish-German, 1997; Nagamine et al., 1997). AIRE is a transcription factor that plays a vital role in maintaining central tolerance and preventing autoimmunity (Anderson and Su, 2016). It is located on chromosome 21q22.3 and consists of 14 exons encoding a 545-amino-acid protein with a molecular weight of 58 kDa (Finnish-German, 1997; Nagamine et al., 1997). AIRE contains four subunit domains, including the caspase recruitment domain/homogeneously staining (CARD/HSR) region, a SAND (SP100, AIRE-1, NucP41/75, DEAF-1) domain, and two plant homeodomain zinc fingers (PHD1, PHD2) (Perniola and Musco, 2014; Perniola, 2018). In addition, there are two NLS (nuclear localization signal) regions, a proline-rich region, and four LXXLL (where L is leucine and X is any amino acid) motifs (Perniola and Musco, 2014; Perniola, 2018). Outside the well-characterized role in immune system, AIRE has been reported to act as a critical spindle-associated protein in embryonic stem (ES) cells (Gu et al., 2017) and has biological functions in stem cell proliferation via promoting self-renewal of ES cells through Lin28 (Bin et al., 2012; Gu et al., 2017).

To date, 170 mutations in the AIRE gene have been reported in the Human Gene Mutation Database *(* http://www.hgmd.cf.ac.uk/ac/gene.php?gene=AIRE). In these mutations, the most common mutation is R257X, which is located in the SAND domain (Husebye et al., 2009; Guo et al., 2018). It is more prevalent in Finnish and Eastern European APS-1 patients. The other major mutation is a 13 base-pair deletion (967-979del13bp) in exon 8, which is common in Norway, British and American Caucasian patients (Husebye et al., 2009; Guo et al., 2018). Additionally, the PHD1 dominant mutants were recently found with relatively high frequency (>0.0008) in mixed populations (Oftedal et al., 2015). In contrast, APS-1 is rare in Chinese populations. Until now, only approximately 20 cases have been reported. In this study, we described a 36-years-old male APS-1 patient who presented with hypoparathyroidism and Addison’s disease. After sequencing, a novel mutation of the AIRE gene (c.1024C>T) was identified. Since this novel mutation was adjacent to PHD1, functional analysis was performed to test the dominant capacity. Our study found that p.Q342X mutant exerted a dominant-negative effect on wildtype (WT) AIRE function.

## 2 Materials and methods

### 2.1 Ethics statement

This study was approved by the Ethics in Research Committee of the First Affiliated Hospital, Zhejiang University (approval number: 2021IIT757). Informed consent was obtained from all individuals involved in the study.

### 2.2 Detection of autoantibodies (Abs) against interferon- α2 and- ω (IFN-α2 and IFN-ω)

Serum samples were serially diluted (1:200; 1:2,000; 1:20,000, and 1:200,000) and incubated with recombinant human IFN-α2 or IFN-ω at a final concentration of 150 pg/ml or 200 pg/ml for 3 h at room temperature. IFN-α2 or IFN-ω levels were determined using a human IFN-α2 or IFN-ω ELISA kit (TSZ, United States) according to the manufacturer’s instructions. Inhibition assays were performed in duplicate for all samples.

### 2.3 Genetic analysis

Blood samples were harvested from all participants and genomic DNA was extracted from peripheral blood leukocytes using RelaxGene Blood DNA System (Tiangen, Beijing, China) in accordance with the manufacturer’s instructions. DNA was dissolved in sterilized double-distilled water and kept at -20 °C until assayed. DNA degradation and contamination were monitored on 1% agarose gel. All DNA samples were examined for protein contamination (as indicated by the A260/A280 ratio) and reagent contamination (indicated by the A260/A230 ratio) with NanoDrop ND 1000 spectrophotometer (NanoDrop, Wilmington, DE).

### 2.4 Mutation identified by Sanger sequencing

Sanger sequencing was performed for the proband and other family members. 13 pairs of primers (Supplementary Table S1) covering the whole exons of AIRE gene were designed upon request. The touch-down PCR was performed in a 30 μl reaction mixture containing 1 μl DNA template, 2 μl forward primer (2.5 μM), 2 μl reverse primer (2.5 μM), 15 μl TaqMix/Taq DNA polymerase and 10 μl double-distilled water. The PCR conditions were: initial denaturation for 5 min at 95°C, 35 cycles of amplification (at 95°C for 30 s, at 65–50°C for 30 s starting from 65°C with decreasing by 1°C every cycle for 15 cycles until remaining at 50°C for 20 cycles, and at 72°C for 30 s), and final elongation at 72°C for 10 min. The amplification products were tested by 2% agarose gel electrophoresis and further analyzed by Sanger sequencing with automated DNA capillary sequencer (3730XL, Applied Biosystems). The sequencing results were analyzed by Mutation Surveyor V3.00.

### 2.5 Construction of WT and mutant AIRE recombination plasmids

AIRE cDNA PCR amplification product was sub-cloned into pEGFP-C1 vector using ClonExpress^®^II (Vazyme, Nanjing, China) to construct recombined plasmid pEGFP-AIRE. Site-directed mutagenesis PCR was used to construct the AIRE mutants. Primers used in site-directed mutagenesis were shown in Supplementary Table S2. Each mutant was achieved by two-step PCRs using pEGFP-AIRE as the template.

For c.1024C>T, two pairs of pEGFP-C1-AIRE-F and pEGFP-C1-AIRE-M1024-R and pEGFP-C1-AIRE-R and pEGFP-C1-AIRE-M1024-F were used in the first PCR step. pEGFP-C1-AIRE-F and pEGFP-C1-AIRE-R were used in the second step. For each mutation, amplification products in the first step were cleaned (Axygen, CA, United States), mixed and used as the template in the second PCR reaction. All final PCR amplifications were recombined into the digested pEGFP-C1 vector using ClonExpress^®^II. Recombinant plasmids pEGFP-AIRE and pEGFP-AIRE-M1024 were transformed into *Escherichia coli* DH5α cells. DNA was prepared using a plasmid DNA purification kit (NucleoBond Xtra midi kit, MN, Germany) according to the manufacturer’s instructions. Sanger sequencing was used to verify sequences. pEGFP-C1-AIRE was cloned into pmCherry-C1 vector by double digestion (BglII and SalⅠ).

### 2.6 Cell culture and transfection

HEK293T cells were cultured in Dulbecco’s Modified Eagle Medium (DMEM) with 10% fetal bovine serum (FBS) at 37°C with 5% CO_2_. 1 µg plasmids DNA with different ratios of WT and mutant AIRE reconstructed vectors were co-transfected into HEK293T cells using PolyJet Transfection reagent (SignaGen Laboratories, MD, United States) according to the manufacturer’s instructions.

### 2.7 Immunofluorescence staining

After incubation for 24 h, transfected cells were fixed using 4% paraformaldehyde for 15 min at room temperature. After fixation, cells were washed three times with PBS. Thereafter, they were incubated with 0.2% Triton X-100 for permeabilization. After washing with PBS, nuclei were stained with DAPI (1 mg/ml). Confocal images were taken with the microscope (Olympus FV3000, Japan).

### 2.8 Western blot analysis

Transfected cells were harvested and lysed with RIPA buffer for 20 min on ice, followed by centrifugation at 12,000 rpm for 20 min at 4°C. Afterwards, protein extracts were separated on a 10% SDS-polyacrylamide gel and electroblotted onto PVDF membranes. After blocking with 5% skimmed milk, the membranes were incubated with GFP-Tag monoclonal antibody (1:2500, ImmunoWay Biotechnology Company, United States) overnight at 4°C. The membranes were then incubated with HRP^*^ goat anti-mouse IgG (H + L) (1:200,000, ImmunoWay Biotechnology Company, United States) for 2 h at room temperature. Finally, the chemiluminescence kit (Biosharp, Anhui) was used to detect the protein signals according to the manufacturer’s protocol.

### 2.9 qRT-PCR analysis

After transfection for 24 h, the cells were lysed with RNAisoTM Plus (Takara, Japan). Chloroform (0.2 ml) was added to the homogenate. The tube was then spun and top aqueous layer was transferred to a new tube. Isopropyl alcohol was added and centrifuged. The pellet was washed with 1 ml of 75% ethanol and vortexed briefly. The tube was then centrifuged again, and the RNA pellet was dried. Afterward, the pellet was dissolved in 100 μl of ultra-pure water and quantified using the NanoDrop 2000 Spectrophotometer. Total RNA (1 μg) was then reverse-transcribed into cDNA in a 20 μl reaction mix using a PrimeScript RTTM reagent Kit (Takara, Japan). Quantitative PCR was performed using PowerUp^TM^ SYBR Green Master Mix (Applied Biosystems, United States). Each sample was tested in triplicate. The primer sequences were listed in Supplementary Table S3.

### 2.10 Statistical analysis

Data were analyzed by ordinary one-way ANOVA using GraphPad Prism v8.0.2. *p* < 0.05 was considered statistically significant.

## 3 Results

### 3.1 Clinical characteristics and identification of AIRE gene mutation

The patient was found to have low blood pressure during physical examination 12 years prior and was not further examined at that time. He was admitted to a local hospital for numbness and convulsions in his limbs 8 years prior and was diagnosed with primary hypoparathyroidism. He was then diagnosed with Addison’s disease in our hospital due to weakness, darkening of the skin and mucous membranes, poor appetite, and low blood pressure 7 years prior. After his diagnosis, he received supplemental therapy with calcium, calcitriol and hydrocortisone. During treatment, he sometimes felt fatigued in his hands and feet. After this hospitalization, he underwent a series of tests, and the results were shown in Table 1. The dose of hydrocortisone was adjusted according to the patient’s clinical symptoms and laboratory results, with the final dose being 20 mg at 8:00 and 10 mg at 16:00. The level of calcium was 2.03 mmol/L, but the patient had no signs or symptoms of hypocalcaemia. Therefore, the doses of calcium and calcitriol were not adjusted (Brandi et al., 2016). In addition, Abs against IFN-α2 and IFN-ω in the proband were tested. The results showed that IFN-α2 Ab was positive, whereas IFN-ω Ab was negative.

**TABLE 1:** Clinical laboratory data of the proband.

	Value	References range
ACTH (8a.m.) (pg/ml)	676.00	0–46.00
COR (8a.m.) (μg/dl)	<1.00	5.00–25.00
Na (mmol/L)	132	136–145
Cl (mmol/L)	94	94–108
PTH (pg/ml)	4.7	15.0–65.0
25(OH)D3 (nmol/L)	80.8	12.3–107.0
Ca (mmol/L)	2.03	2.03–2.54
P (mmol/L)	1.84	0.87–1.45
FBG (mmol/L)	4.55	3.90–6.10
FSH (mIU/ml)	2.6	1.4–18.1
LH (mIU/ml)	6.08	1.50–9.30
T (ng/dl)	555.8	241.0–827.0
TSH (mIU/L)	1.846	0.380–4.340
FT4 (pmol/L)	20.08	10.45–24.38
IFN-ω Ab	Negative	Negative
IFN-α2 Ab	Positive	Negative

ACTH, Adrenocorticotropic hormone; COR, Cortisol; PTH, Parathyroid hormone; FBG, Fasting blood glucose; FSH, Follicle-Stimulating Hormone; LH, Luteinizing hormone; T, Testosterone; TSH, Thyroid Stimulating hormone; FT4, Free thyroxine; IFN, Interferon.

He was diagnosed with APS-1 according to the clinical diagnostic criteria of APS-1. However, he did not suffer from chronic mucocutaneous candidiasis and other autoimmune diseases, including autoimmune thyroiditis, type I diabetes, autoimmune hepatitis, and vitiligo.

**FIGURE 1:**
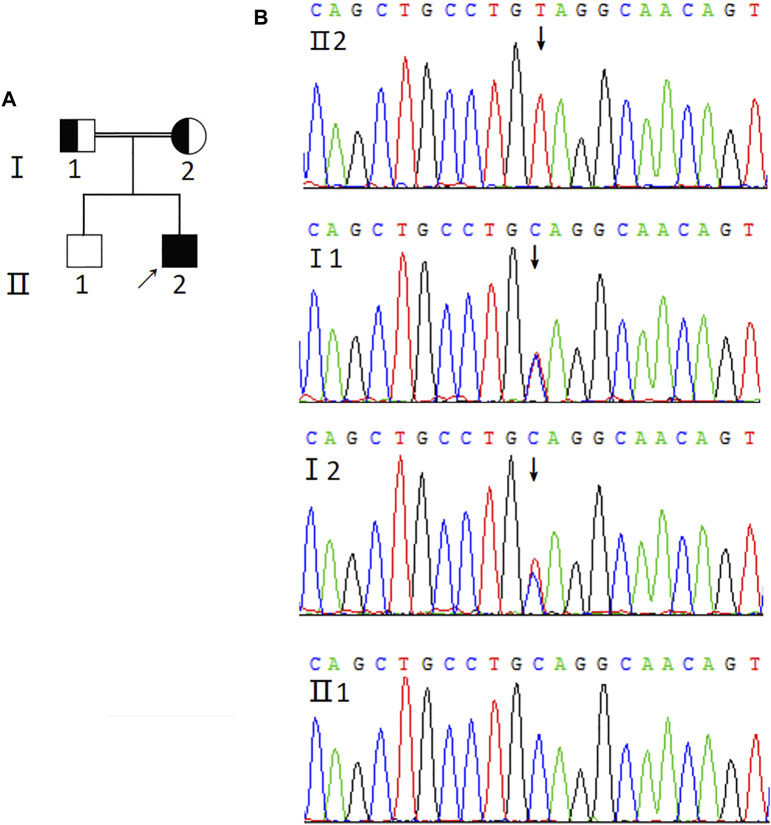
Genetic analysis of AIRE mutation. **(A)** Pedigree of the APS-1 family. Solid symbol indicates the affected individuals, open symbol belongs to the unaffected individuals, square represents male and circle represents female. Arrow indicates the proband (Ⅱ2). The half-shaded icons denote the mutation carriers. **(B)** Sequencing profiles of the pedigree. The sequencing result of the proband (Ⅱ2) shows the homozygous mutation (c.1024C>T) in exon 9 of the AIRE gene (GenBank Accession: NM_000383.3) indicated by the arrow. The proband’s parents carry the heterozygous variant of the AIRE gene and his older brother is wildtype.

Next, sequencing was performed to identify the AIRE mutation sites. The results revealed a novel homozygous mutation of the AIRE gene (c.1024C>T) in the proband (Figure 1). Additionally, his parents were found to carry the same heterozygous variant of the AIRE gene. However, his older brother was wildtype. Of note, the proband’s parents were in a consanguineous marriage. Unfortunately, the proband’s parents did not undergo further testing for special reasons, so it was unclear whether they had organ-specific autoimmune diseases.

### 3.2 p.Q342X mutant exerted a dominant-negative effect on WT AIRE function

**FIGURE 2:**
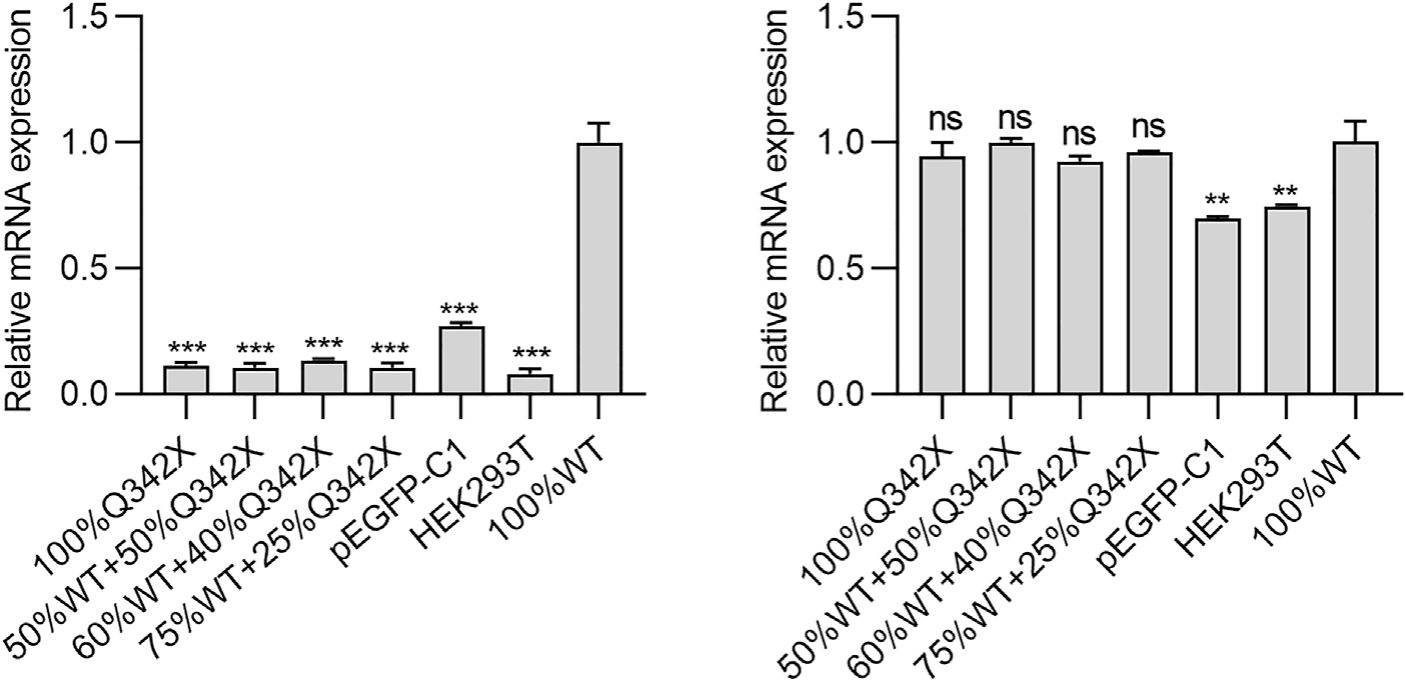
The AIRE- dependent (KRT14 and S100A8) and -independent (PRMT3 and CCNH) genes were tested. Cells were transfected with various amounts of WT AIRE and p.Q342X mutant, alone or in combinations. Thereafter, the mRNA expressions of KRT14, S100A8, PRMT3, and CCNH were examined using qRT-PCR. The results were shown as relative mRNA expression compared to cells transfected only with WT AIRE. *p < 0.05, **p < 0.01, ***p < 0.001.

Oftedal *et al.* reported that several missense PHD1 mutations suppressed gene expression driven by WT AIRE in a dominant-negative manner (Oftedal et al., 2015). Given that p.Q342X mutant is close to PHD1 domain, we speculate whether this mutation would also exert a similar dominant-negative effect. To this end, HEK293T cells transfected with either WT-AIRE and/or p.Q342X mutant expression vectors were used. We then tested the mRNA expression of a panel of AIRE - dependent (keratin 14- KRT14 and S100 calcium binding protein A8-S100A8) and - independent (protein arginine methyltransferase 3, PRMT3 and cyclin H-CCNH) genes. As expected, p.Q342X mutant diminished the expression of AIRE-dependent genes (Figure 2). Surprisingly, when HEK293T cells were co-transfected with different ratios of WT AIRE and p.Q342X mutant, p.Q342X mutant significantly diminished the ability of WT AIRE and p.Q342X mutant. p.Q342X mutant significantly abrogated the ability of WT AIRE to induce the expression of KRT14 and S100A8 (Figure 2). These results indicate that p.Q342X mutant exerted a dominant-negative effect on WT AIRE function.

### 3.3 c.1024C>T mutation caused the truncation of the AIRE protein

**FIGURE 3:**
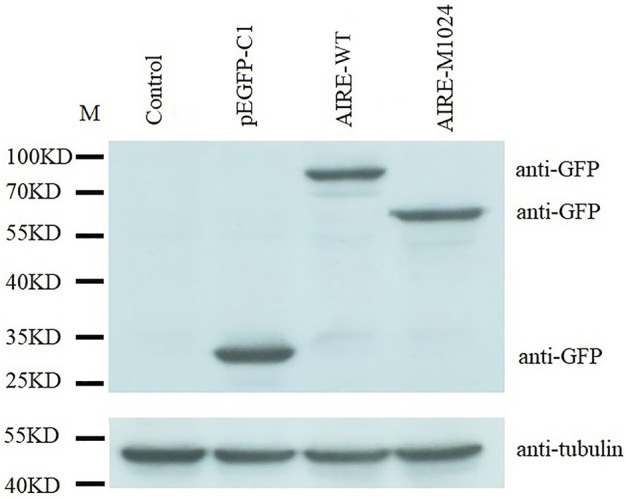
Western blot analysis of HEK293T cells transfected with WT-AIRE or p.Q342X mutant constructs. The bands were blotted with anti-GFP antibody. Tubulin was used as an internal control. Compared with WT AIRE, the p.Q342X mutant translated a shortened product. M: protein marker.

The c.1024C>T mutation of the AIRE gene was predicted to generate a truncation protein. To confirm this prediction, HEK293T cells were transfected with WT-AIRE or p.Q342X mutant expression vectors. Western blot analysis showed that the molecular weight of WT-AIRE protein was 58 kDa. Since the detected band contained the GFP protein (27 kDa), its molecular weight was shown to be 85 kDa. The molecular weight of p.Q342X mutant was 63 kDa in Figure 3. The actual molecular weight was 36kD after removing GFP protein, in agreement with the calculated weight of the mutant. It demonstrated that the c.1024C>T mutation leaded to the truncation of the AIRE protein.

### 3.4 p.Q342X mutant co-localized with WT AIRE in nuclear speckles

**FIGURE 4:**
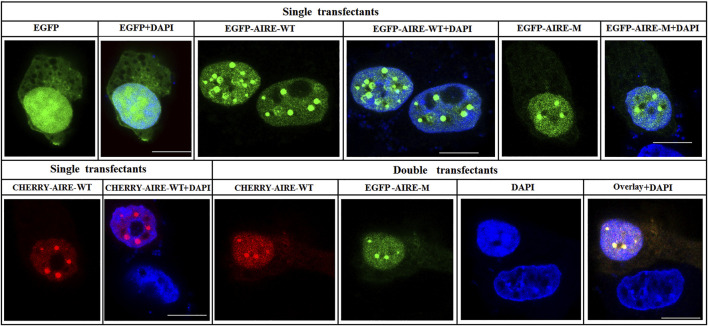
Subcellular localization of p.Q342X mutant. Confocal images showed the subcellular localization of WT AIRE and p.Q342X mutant. Overlay images showed the degree of co-localization (yellow). Nuclei were visualized with DAPI counterstain (blue). The scale bar is 10 μm.

To further understand the property of p.Q342X mutant, immunofluorescent staining was performed to analyze the nuclear localization pattern. HEK293T cells were co-transfected with Cherry-tagged WT AIRE plasmids together with expression vectors encoding p.Q342X mutant tagged with EGFP. Our results revealed that p.Q342X mutant localized in nuclear speckles. Moreover, it co-localized with WT AIRE protein (Figure 4), suggesting that its nuclear localization pattern is not disrupted.

## 4 Discussion

APS-1 is an autosomal recessive disease caused by mutations of the AIRE gene. AIRE is mainly expressed in medullary thymic epithelial cells (mTECs), but it is also present within both the lymph nodes and spleen (Heino et al., 2000). As a vital transcription factor, it functions by regulating the expression of tissue-restricted antigens in mTECs. Subsequently, the T cells that respond to those proteins (autoreactive) are subject to negative selection and undergo apoptosis (Taniguchi and Anderson, 2011). Pathogenic variants in AIRE cause a failure to eliminate autoreactive T cells in the thymus, resulting in the autoimmune manifestations seen in APS-1 (Akirav et al., 2011; Bruserud et al., 2016). In addition, recent studies have revealed AIRE to be involved in the functions of stem cells. Gu *et al.* identified the expression of AIRE in ES cells and showed that the expression of AIRE decreased with the differentiation of ES cells (Gu et al., 2010). Moreover, AIRE has been found to promote the expression of the pluripotent factor Lin28 and the self-renewal of ES cells (Bin et al., 2012).

APS-1 frequently occurs in relatively isolated populations, such as Iranian Jews (1:9,000) (Zlotogora and Shapiro, 1992), Sardinians (1:14,400) (Rosatelli et al., 1998), and Finns (1:25,000) (Betterle et al., 1998). However, it is quite rare in other populations, such as Chinese populations. Its main clinical features include chronic mucocutaneous candidiasis, hypoparathyroidism, and adrenal insufficiency. In addition, other autoimmune diseases may appear concomitantly, including type 1 diabetes, autoimmune hepatitis, premature ovarian insufficiency, and autoimmune thyroid disease.

Chronic mucocutaneous candidiasis is thought to be the primary and most common clinical manifestation in APS-1 patients. Nevertheless, the APS-1 patient we studied only presented with hypoparathyroidism and Addison’s disease, and did not suffer from chronic mucocutaneous candidiasis. This result was in agreement with that reported by Zhu *et al.* from a Chinese population (Zhu et al., 2017). However, the mutation site of the AIRE gene was different between them. In Zhu’s study, the mutation site (c.206A>C) was located in exon 2, and the mutation disrupted the function of the CARD/HSR domain, affecting homodimerization (Zhu et al., 2017). The mutation site of the AIRE gene in our study was located in exon 9 and this homozygous mutation introduced the premature stop codon at the 342^nd^ amino acid, resulting in the truncation of the AIRE protein, as shown in Figure 3. As known, the AIRE protein consists of 545 amino acids, of which the 342^nd^ amino acid-glutamine, is located just after the PHD1 domain (Guo et al., 2018). This mutation caused the loss of the PHD2 domain, a proline-rich region and two LXXLL motifs of the AIRE protein. It has been demonstrated that PHD2 influences the ability of AIRE to regulate the medullary epithelial cell transcriptome (Yang et al., 2013; Perniola and Musco, 2014). LXXLL motifs are shown to be involved in the process of gene transcription (Perniola and Musco, 2014). Therefore, it is speculated that this mutation may affect cell transcription and lead to autoimmunity.

Next, we investigated the property of p.Q342X mutant *in vitro*. Morphological results showed that the p.Q342X mutant localized in nuclear speckles and co-localized with WT AIRE, which was consistent with another study from Husebye’s group (Oftedal et al., 2015). It is known that the CARD domain plays an important role in the specked nuclear localization of AIRE (Bjorses et al., 1999), whereas p.Q342X mutant was behind PHD1 domain, therefore it did not disrupt AIRE’s nuclear localization. Previous study showed that mono-allelic mutations with dominant effects clustered within the PHD1 domain of AIRE(Oftedal et al., 2015). Strikingly, p.Q342X mutant in our study also exerted a dominant-negative effect on WT AIRE. It suggested that mutations near the PHD1 domain might also exert a similar effect. A recent study found that the dominant mutations augmented the expression of dysfunctional AIRE with an altered capacity to bind chromatin and induce gene expression (Goldfarb et al., 2021). Whether p.Q342X mutant also acts through a similar mechanism remains to be further studied. In addition, Oftedal *et al.* found that truncating AIRE mutations such as p.R257^*^ and p.C311^*^ manifested in a recessive manner (Oftedal et al., 2015). However, it is rather surprising that p.Q342X mutant behaved in a dominant-negative manner. Moreover, IFN-α2 Ab was positive in the proband, whereas IFN-ω Ab was negative. The underlying mechanism is not clear, and may be related to different mutation sites.

There are some limitations in our study. First, the proband’s parents are not further examined, so it is unknown if they have organ-specific autoimmune diseases. Second, formal proofs of dominant-negative effect are few, and further studies need to be investigated in the future.

## 5 Conclusion

c.1024C>T is a mutation in exon 9 of the AIRE gene that was reported for the first time. Furthermore, our studies find that the mutation in the other part of AIRE gene is able to also exert a dominant-negative effect in addition to SAND, PHD1 and PHD2 domains (Cetani et al., 2001; Oftedal et al., 2015; Goldfarb et al., 2021). This study enriched the AIRE mutation database and contributed to further understanding of APS-1.

## Data Availability

The raw data supporting the conclusion of this article will be made available by the authors, without undue reservation.
