# Supplementary material for: A novel AIRE mutation leads to autoimmune polyendocrine syndrome type-1
Source: Front Cell Dev Biol. 2022 Aug 22;10:948350. doi: 10.3389/fcell.2022.948350 (PMC9441485; doi:10.3389/fcell.2022.948350)
Supplement: Supplementary file 1 [file DataSheet1.docx]

**Supplementary Tables S1-S3**

**Supplementary Table S1. The primers for whole exons of AIRE gene.**

| **Name** | **Forward primer (5’-3')** | **Reverse primer (5’-3')** |
| --- | --- | --- |
| AIRE-E1 | CCTCCATCACAGGGAAGTGT | GGGAAGGGGACCAACTTCTA |
| AIRE-E2 | TACACCACCACCTGACTCCA | CAACCTCAGGGTTTTCTCCA |
| AIRE-E3 | GAACCGGAGTGGTGTTTGAG | TGGTCCAGTGTGTGGGTCTA |
| AIRE-E4 | ACACTGGACCAGCCTCTCAG | TGCTTAGACCCAAGGTGTCC |
| AIRE-E5 | CTTACCTGTGGGCTGTCTGC | ACCACAGGCAGAAACTCTGG |
| AIRE-E6E7 | GGAACTCCACCTGTCTCTGC | AGCTGTACCCTGTGGGTAGG |
| AIRE-E8 | GTTGGAGACCAGATGGATGG | TTCCATCTTGGATGGGAGAG |
| AIRE-E9 | CTCCCTTCCTGTGTCTCTGC | GCCAGGAGAGCTGGGTTTAG |
| AIRE-E10 | TTCCTTGTTCTGCTGCTGTG | CCTTCATTGTCCTGCCTTGT |
| AIRE-E11 | CACCGCCTTTCAGGAGACT | GTGTGGTTGTGGGCTGTATG |
| AIRE-E12 | CTCATACCCTGCACCTCACC | GTCTGCCCTGAGATGTGCTC |
| AIRE-E13 | CCAGTGGAGCTGGGTGTAAG | CTGAGTTTCCACGGCTCAAG |
| AIRE-E14 | CCTTTGATGGAATACGGTGAA | TAGTAGGTCACCAGGCAAGGA |

**Supplementary Table S2. The primers used in site-directed mutagenesis.**

| **Name** | **Forward primer (5’-3')** | **Reverse primer (5’-3')** |
| --- | --- | --- |
| pEGFP-C1-AIRE | CGAGCTGTACAAGTCCGGACTCAGATCTCGAGGCATGGCGACGGACGCGGCGCTACGCCGGCT | CGGGCCCGCGGTACCGTCGACTGCAGAATTCTCAGGAGGGGAAGGGGGCCGCCGGACGGGCCAT |
| pEGFP-C1-AIRE-M1024 | GCTCCAGCTGCCTGTAGGCAACAGTCCAGG | CCTGGACTGTTGCCTACAGGCAGCTGGAGC |

**Supplementary Table S3. The primers used in qRT-PCR analysis.**

| **Name** | **Forward primer (5'-3')** | **Reverse primer (5'-3')** |
| --- | --- | --- |
| PRMT3  CCNH | TCCAGAAGCTGTTGTGGAAGT  GCATTGACGGATGCTTACCT | CAGTGTGTTTTGGTGCTCTGA  TGACATCGCTCCAACTTCTG |
| KRT14  S100A8 | GACCATTGAGGACCTGAGGA  ATGCCGTCTACAGGGATGAC | GGCTCTCAATCTGCATCTCC  TGGCTTTCTTCATGGCTTTT |
| B2M | AGGCTATCCAGCGTACTCCA | CGGCAGGCATACTCATCTTT |
